# Recent Advances in Nanotechnology Applied to Biosensors

**DOI:** 10.3390/s90201033

**Published:** 2009-02-17

**Authors:** Xueqing Zhang, Qin Guo, Daxiang Cui

**Affiliations:** Department of Bio-Nano Science and Engineering, Key Laboratory for Thin Film and Microfabrication Technology of Ministry of Education, National Key Laboratory of Micro /Nano Fabrication Technology, Research Institute of Micro/Nano Science and Technology, Shanghai Jiao Tong University, Shanghai, 200240, P.R. China; E-Mails: zhangxueqing@gmail.com (X. Z.); guoqin1985@msn.com (G. Q)

**Keywords:** Biosensor, nanotechnology, gold nanoparticle, carbon nanotubes, quantum dots, magnetic nanoparticles

## Abstract

In recent years there has been great progress the application of nanomaterials in biosensors. The importance of these to the fundamental development of biosensors has been recognized. In particular, nanomaterials such as gold nanoparticles, carbon nanotubes, magnetic nanoparticles and quantum dots have been being actively investigated for their applications in biosensors, which have become a new interdisciplinary frontier between biological detection and material science. Here we review some of the main advances in this field over the past few years, explore the application prospects, and discuss the issues, approaches, and challenges, with the aim of stimulating a broader interest in developing nanomaterial-based biosensors and improving their applications in disease diagnosis and food safety examination.

## 1. Introduction

A biosensor is a device incorporating a biological sensing element either intimately connected to or integrated within a transducer. Specific molecular recognition is a fundamental prerequisite, based on affinity between complementary structures such as enzyme-substrate, antibody-antigen and receptor-hormone, and this property in biosensor is used for the production of concentration–proportional signals. Biosensor's selectivity and specificity highly depend on biological recognition systems connected to a suitable transducer [1-3].

In recent years, with the development of nanotechnology, a lot of novel nanomaterials are being fabricated, their novel properties are being gradually discovered, and the applications of nanomaterials in biosensors have also advanced greatly. For example, nanomaterials-based biosensors, which represent the integration of material science, molecular engineering, chemistry and biotechnology, can markedly improve the sensitivity and specificity of biomolecule detection, hold the capability of detecting or manipulating atoms and molecules, and have great potential in applications such as biomolecular recognition, pathogenic diagnosis and environment monitoring [4-6].

Here we review some of the main advances in this field over the past few years, explore the application prospects, and discuss the issues, approaches, and challenges, with the aim of stimulating a broader interest in developing nanomaterials-based biosensor technology.

## 2. The Use of Nanomaterials in Biosensors

To date, modern materials science has reached a high degree of sophistication. As a result of continuous progress in synthesizing and controlling materials on the submicron and nanometer scales, novel advanced functional materials with tailored properties can be created. When scaled down to a nanoscale, most materials exhibit novel properties that cannot be extrapolated from their bulk behavior. The interdisciplinary boundary between materials science and biology has become a fertile ground for new scientific and technological development. For the fabrication of an efficient biosensor, the selection of substrate for dispersing the sensing material decides the sensor performance. Various kinds of nanomaterials, such as gold nanoparticles [7], carbon nanotubes (CNTs) [8], magnetic nanoparticles [9] and quantum dots [10], are being gradually applied to biosensors because of their unique physical, chemical, mechanical, magnetic and optical properties, and markedly enhance the sensitivity and specificity of detection.

### 2.1. The Use of Gold Nanoparticles in Biosensors

Gold nanoparticles (GNPs) show a strong absorption band in the visible region due to the collective oscillations of metal conduction band electrons in strong resonance with visible frequencies of light, which is called surface plasmon resonance (SPR). There are several parameters that influence the SPR frequency. For example, the size and shape of nanoparicles, surface charges, dielectric constant of surrounding medium etc. By changing the shape of gold nanoparticles from spherical to rod, the new SPR spectrum will present two absorption bands: a weaker short-wavelength in the visible region due to the transverse electronic oscillation and a stronger long-wavelength band in NIR due to the longitudinal oscillation of electrons. The change of aspect ratio can greatly affect the absorption spectrum of gold nanorods (GNRs) [11]. In the same vein, increasing the aspect ratio can lead to longitudinal SPR absorption band redshifts. Different GNP structures shows different properties. In comparison with a gold nanoparticle-conjugating probe, the gold nanowire-functionalized probe could avoid the leakage of biomolecules from the composite film, and enhanced the stability of the sensor [12,13]. This interesting phenomenon will be enormously beneficial in practical applications such as biosensors.

**Figure 1.**
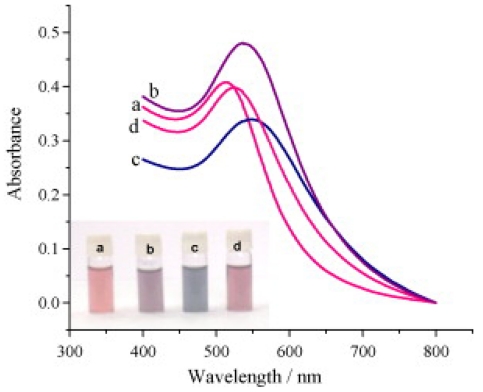
Absorption spectra illustrating the protamine-induced aggregation and heparin-driven de-aggregation of AuNPs. (a) AuNPs alone; (b, c) after the addition of protamine: (b) 0.7 μg/ml and (c) 1.6 μg/ml; (d) after the addition of heparin (10.2 μg/mL). Inset shows the corresponding colorimetric response [14].

It is well known that well-dispersed solutions of GNPs display a red color, while aggregated GNPs appear a blue color. Based on this phenomenon, Jena *et al.* [14] established a GNPs-based biosensor to quantitatively detect the polyionic drugs such as protamine and heparin. As shown in Figure 1, the degree of aggregation and de-aggregation of GNPs is proportional to the concentration of added protamine and heparin.

**Figure 2.**
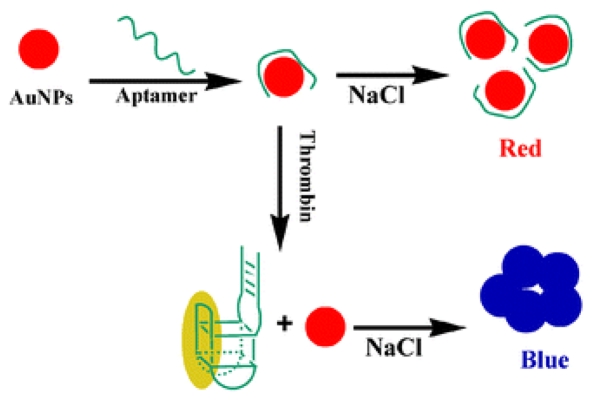
AuNPs colorimetric strategy for thrombin detection [16].

Non-crosslinking GNP aggregation can also be applied for enzymatic activity sensing and potentially inhibitor screening [15]. Wei *et al.* [16] described a simple and sensitive aptamer-based colorimetric sensing of alpha-thrombin protein using unmodified 13 nm GNP probes, as shown in Figure 2. This method's advantage lies in that the general steps such as surface modification and separation can be avoided, which ensures the original conformation of the aptamer while interacting with its target, thereby leading to high binding affinity and sensitive detection.

GNPs in biosensors can also provide a biocompatible microenvironment for biomolecules, greatly increasing the amount of immobilized biomolecules on the electrode surface, and thus improving the sensitivity of the biosensor [17, 18]. The glassy carbon electrode (GCE) was widely used in biosensor, and GNP modified GCEs showed much better electrochemical stability and sensitivity. GNPs and methylene blue (MB) could be assembled via a layer-by-layer (LBL) technique into films on the GCE modified for detection of human chorionic gonadotrophin (HCG) [19]. Due to the high surface area of the nanoparticles for loading anti-HCG, this immunosensor can be used to detect the HCG concentration in human urine or blood samples.

For the detection of reduction of H_2_O_2_, GNP-modified electrodes also showed much wider pH adaptive range and larger response currents [20]. Due to the large specific surface area and good biocompatibility of GNPs, horseradish peroxidase (HRP) can be adsorbed onto a GNP layer for the detection of H_2_O_2_ without loss of biological activity [21]. Shi *et al.* [22] confirmed that this kind of HRP-GNP biosensor exhibited long-term stability and good reproducibility.

**Figure 3.**
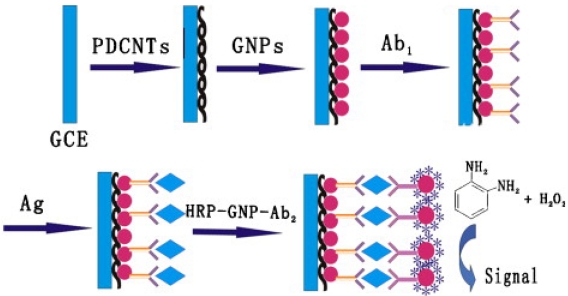
The immunoassay procedure of GNPs/PDCNTs modified immunosensor using HRP–GNPs–Ab_2_ conjugates as label [24].

GNPs/CNTs multilayers can also provide a suitable microenvironment to retain enzyme activity and amplify the electrochemical signal of the product of the enzymatic reaction [23]. For example, GNPs/CNTs nanohybrids were covered on the surface of a GCE, which formed an effective antibody immobilization matrix and gave the immobilized biomolecules high stability and bioactivity. The approach provided a linear response range between 0.125 and 80 ng/mL with a detection limit of 40 pg/mL. As shown in Figure 3, because of the advantages of GNPs and CNTs, the hybrid composite has more potential applications for electrochemical sensor, which could be easily extended to other protein detection schemes and DNA analysis [24]. For example, Wang *et al.* [25] described the fabrication of ZrO_2_/Au nano-composite films through a combination of sol–gel procedure and electroless plating, the organophosphate pesticides (Ops) can be strongly adsorped on the ZrO_2_/Au film electrode surface, which provides an effective quantitative method for OPs analysis.

The gold nanorods (GNR) modified electrode layer shows a better analytical response than GNPs [26]. GNR based immunosensors have advantages such as simplicity, being label free, low sample volume, reusability and being more suitable for lab-on-chip devices over gold nanoparticles. GNRs are sensitive to the dielectric constant of the surrounding medium due to surface plasmon resonance, therefore a slight change of the local refractive index around GNRs will result in an observable plasmon resonance frequency shift. Irudayaraj and Yu fabricated different aspect ratios of GNRs with targeted antibodies to detect three targets (goat anti-human IgG1 Fab, rabbit antimouse IgG1 Fab, rabbit anti-sheep IgG (H+L)). Results showed that GNRs can be used for a multiplexing detection device of various targets. In another study, they examined the quantification of the plasmonic binding events and estimation of ligand binding kinetics tethered to GNRs via a mathematical method. The GNRs sensors were found to be highly specific and sensitive with a dynamic response in the range between 10^-9^ M and 10^-6^ M. For higher-target affinity pair, one can expect to reach femtomolar levels limit of detection. This is promising for developing sensitive and precise sensors for biological molecule interactions. Chilkoti and his co-workers have miniaturized the biosensor to the dimensions of a single gold nanorod [27]. Based on a proof-of-concept experiment with streptavidin and biotin, they tracked the wavelength shift using a dark-field microspectroscopy system. GNRs binding 1 nM of streptavidin could bring about a 0.59 nm mean wavelength shift. Furthermore, they also indicated that the current optical setup could reliably measure wavelength shifts as small as 0.3 nm. Frasch and co-workers have set single molecules DNA detection in spin by linking F1-ATPase motors and GNRs[28]. The biosensor overcomes the defects inherent to PCR or LCR, is faster and reaches zeptomol concentrations, which is greatly superior to traditional fluorescence-based DNA detection systems which have only about a 5 picomolar detection limit.

### 2.2. The Use of CNTs in Biosensors

**Figure 4.**
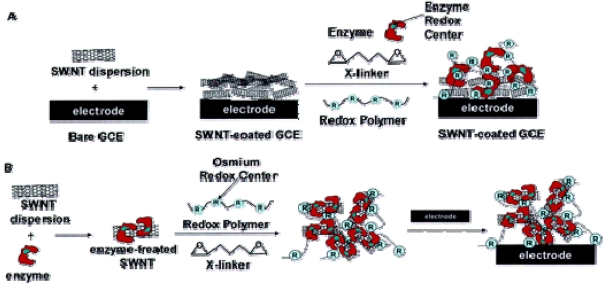
Schematic of the construction of type A and type B sensors. (A) Fabrication of type A sensors in which a film of SWNTs was first cast onto a bare glassy carbon electrode and allowed to dry, before an alquot of the redox hydrogel was cast on top of the SWNT-coated electrode. (B) Fabrication of type B sensors in which SWNTs were first incubated with an enzyme solution before they were incorporated into the redox hydrogel. An aliquot of the redox hydrogel solution containing the enzyme-modified SWNTs was then cast on top of a bare glassy carbon electrode [31].

**Figure 5.**
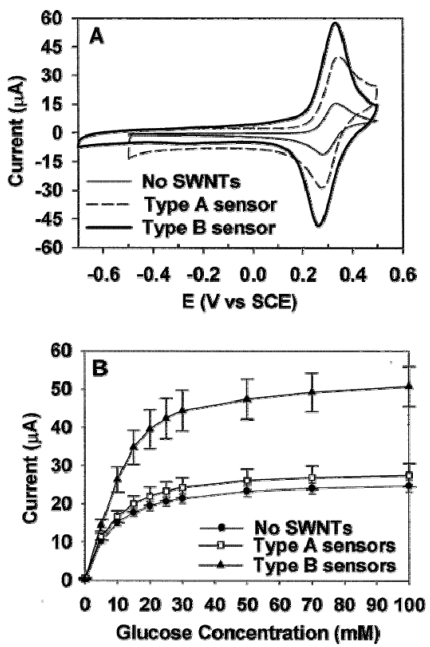
Electrochemical characterization of glucose oxidase sensors. (A) Cyclic voltammograms of a GCE modified with the redox hydrogel alone (-); a GCE modified first with a film of SWNT and then coated with the redox hydrogel (----) (type A sensor); (III) a GCE modified with a redox hydrogel containing GOX-treated SWNTs (-) (type B sensor). Scan rate 50 mV/s. (B) Glucose calibration curves for the three types of sensors described in (A). T = 25C, E = 0.5 V vs SCE. Values are mean ±SEM [31].

Since Iijima discovered carbon nanotubes (CNTs) in 1991, CNTs have attracted enormous interest due to their many novel properties such as unique mechanical, physical, chemical properties. CNTs have great potential in applications such as nanoelectronics, biomedical engineering, and biosensing and bioanalysis [5, 29, 30]. For example, polymer-CNTs composites can achieve high electrical conductivity and good mechanical properties, which offer the exciting possibility of developing ultrasensitive, electrochemical biosensors. As shown in Figure 4 and Figure 5, amperometric biosensors [31] was constructed by incorporation of single-walled carbon nanotubes modified with enzyme into redox polymer hydrogels. First, an enzyme was incubated in a single-walled carbon nanotube (SWNT) solution, then cross-linked within a poly[(vinylpyridine)Os(bipyridyl)(2)Cl^2+/3+^] polymer film, and finally formed into composite films. The redox polymer films incorporated with glucose oxidase modified SWNTs resulted in a 2 to 10-fold increase in the oxidation and reduction peak currents during cyclic voltammetry, while the glucose electrooxidation current was increased 3-fold to close to 1 mA/cm2 for glucose sensors. Similar effects were also observed when SWNTs were modified with horseradish peroxidase prior to incorporation into redox hydrogels.

Conductive polymer-based nano-composite has been utilized as a MEMS sensing material via a one-step, selective on-chip deposition process at room temperature [32]. For example, the doped PPy-MWCNT is confirmed to be sensitive to glucose concentrations up to 20 mM, which covers the physiologically important 0-20 mM range for diabetics, so they can be used for diagnosis of diabetes [33, 34]. So far, these electrochemical sensors such as enzyme-based biosensors, DNA sensors and immunosensors have been developed based on polymer-CNT composites, and can be used to diagnose different kinds of diseases quickly [35, 36].

The bionanocomposite layer of multiwalled carbon nanotubes (MWNT) in chitosan (CHIT) can be used in the detection of DNA [34]. The biocomponent, represented by double-stranded herring sperm DNA, was immobilized on this composite using layer-by-layer coverage to form a robust film. SsDNA probes could be immobilized on the surface of GCE modified with MWNTs/ZnO/CHIT composite film [37]. The sensor can effectively discriminate different DNA sequences related to PAT gene in the transgenic corn, with a detection limit of 2.8 mol/L of target molecues.

Carbon nanofibers are found to be an effective strategy for building a biosensor platform [38]. Bai *et al.* [39] found that the synergistic effects of MWNTs and ZnO improved the performance of the biosensors formed. They reported an amperometric biosensor for hydrogen peroxide, which was developed based on adsorption of horseradish peroxidase at the GCE modified with ZnO nanoflowers produced by electrodeposition onto MWNTs film. Zhang *et al.* described a controllable layer-by-layer self-assembly modification technique of GCE with MWNTs and introduce a controllable direct immobilization of acetylcholinesterase (AChE) on the modified electrode. By the activity decreasing of immobilized AChE caused by pesticides, the composition of pesticides can be determined [40-43].

**Figure 6.**
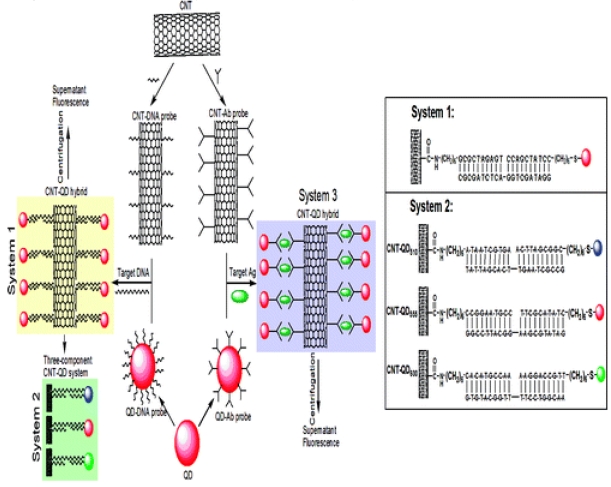
Surface functionalization of CNT (or QD) with oligonucleotide/Angibody (Ab), forming CNT-DNA (or -Ab) probe and QD-DNA (or-Ab) probe, and subsequent addition of target oligonucleotide (or Antigen) to form CNT-QD assembly. The unbound QD probe was obtained by simple centrifugation separation and the supernatant fluorescence intensity of QDs was monitored by spectrofluorometer. (System 1) Formation of CNT-QD hybrid in the presence of complementary DNA target; (System 2) Three-component CNT-QD system with the purpose to detect three different DNA target simultaneously; (System 3) CNT-QD protein detection system based on antigen-antibody immunoreactions [44].

Our group also just reported a highly selective, ultrasensitive, fluorescent detection method for DNA and antigen based on self-assembly of multi-walled carbon nanotubes (CNT) and CdSe quantum dots (QD) via oligonucleotide hybridization; its principle is shown in Figure 6 [44]. Multi-walled carbon nanotubes (CNTs) and QDs, their surfaces are functionalized with oligonucleotide(ASODN) or antibody (Ab), can be assembled into nanohybrid structures upon the addition of a target complementary oligonucleotide or antigen (Ag). As shown in Figure 6, nanomaterial building blocks that vary in chemical composition, size or shape are arranged in space on the basis of their interactions with complementary linking oligonucleotide for potential application in biosensors. We show how this oligonucleotide directed assembly strategy could be used to prepare binary (two-component) assembly materials comprising two different shaped oligonucleotide-functionalized nanomaterials. Importantly, the proof-of-concept demonstrations reported herein suggest that this strategy could be extended easily to a wide variety of multicomponent systems.

### 2.3. The Use of Magnetic Nanoparticales in Biosensor

Magnetic nanoparticles (MNP), because of their special magnetic properties, have been widely explored in applications such as hyperthermia [45], magnetic resonance imaging (MRI) contrast agent [46], tissue repair [47], immunoassay [48], drug/gene delivery [49], cell separation [50], GMR-sensor [51], etc. Zhang *et al.* [52] prepared a new kind of magnetic dextran microsphere (MDMS) by suspension crosslinking using iron nanoparticles and dextran. HRP was then immobilized on a MDMS-modified GCE. On the basis of the immobilized HRP-modified electrode with hydroquinone (HQ) as mediator, an amperometric H_2_O_2_ biosensor was fabricated. Lai *et al.* [53] prepared a magnetic chitosan microsphere (MCMS) using carbon-coated MNPs and chitosan. Hemoglobin (Hb) was successfully immobilized on the surface of MCMS modified GCE with the cross-linking of glutaraldehyde.

Janssen *et al.* [54] demonstrated that a rotating magnetic field can be used to apply a controlled torque on superparamagnetic beads which leads to a tunable bead rotation frequency in fluid and develop a quantitative model, based on results from a comprehensive set of experiments. This control of torque and rotation will enable novel functional assays in bead-based biosensors.

The amperometric biosensor was based on the reaction of alkaline phosphatase (ALP) with the substrate ascorbic acid 2-phosphate (AA2P), where the Fe_3_O_4_ nanoparticles have led to the enhancement of the biosensor response with an improved linear response range. This biosensor was applied to the determination of the herbicide 2, 4-dichlorophenoxyacetic acid (2, 4-D) [55].

In fact, a wide variety of methods have been developed for sensing and enumerating individual micron-scale magnetic particles [56]. Direct detection of magnetic particle labels includes Maxwell bridge, Frequency-dependent magnetometer, Superconducting quantum interference device (SQUID) and methods of magnetoresistance. Indirect detection includes Micro-cantilever-based Force Amplified Biological Sensor (FABS) and Magnetic Relaxation Switches (MRS). Two examples follow.

Recently, a highly sensitive, giant magnetoresistance-spin valve (GMR-SV) biosensing device with high linearity and very low hysteresis was fabricated by photolithography [57]. The signal from even one drop of human blood and nanoparticles in distilled water was sufficient for their detection and analysis.

For the immunomagnetic detection and quantification of the pathogen Escherichia coli O157:H7, a giant magnetoresistive multilayer structure implemented as sensing film consists of 20[Cu5.10 nm/Co2.47 nm] with a magnetoresistance of 3.20% at 235 Oe and a sensitivity up to 0.06 Ω/Oe between 150 Oe and 230 Oe. Silicon nitride has been selected as optimum sensor surface coating. In order to guide the biological samples, a microfluidic network made of SU-8 photoresist and 3D stereolithographic techniques have been included [58, 59].

### 2.4. The Use of QDs in Biosensors

Quantum dots have been subject to intensive investigations because of their unique photoluminescent properties and potential applications [60-62]. So far, several methods have been developed to synthesize water-soluble quantum dots for use in biologically relevant studies. For example, quantum dots have been used successfully in cellular imaging [63], immunoassays[64], DNA hybridization [65], biosensor, and optical barcoding [66]. Quantum dots also have been used to study the interaction between protein molecules or detect the dynamic course of signal transduction in live cells by Fluorescence Resonance Energy Transfer (FRET) [67, 68]. These synthesized quantum dots have significant advantages over traditional fluorescent dyes, including better stability, stronger fluorescent intensity, and different colors, which are adjusted by controlling the size of the dots [64]. Therefore, quantum dots provide a new functional platform for bioanalytical sciences and biomedical engineering.

**Figure 7.**
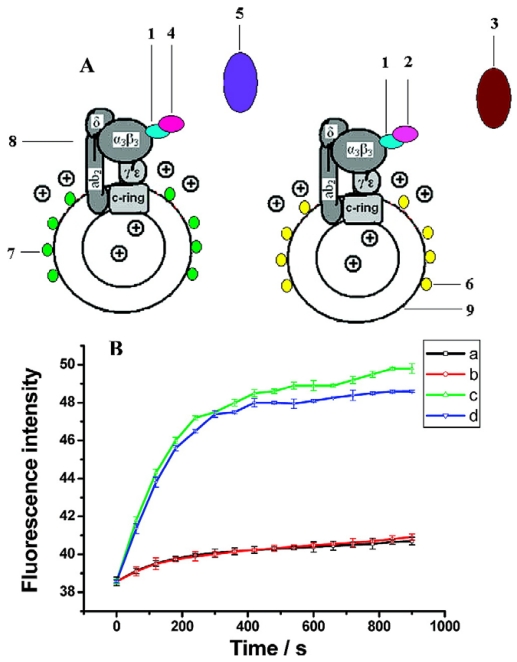
(a) Basic design of QD biosensors based on F0F1-ATPase: (1) antibody of β-subunit; (2) the antibody of MHV68; (3) MHV68; (4) the antibody of H9 avian influenza virus; (5) H9 avian influenza virus; (6) CdTe QDs with emission wavelength at 585 nm; (7) CdTe QDs with emission wavelength at 535 nm; (8) F0F1-ATPase within chromatophores; (9) chromatophores. (b) Changes of fluorescence intensity of QD biosensors with and without viruses. Curve a: The changes of fluorescence intensity of orange QD biosensors without MHV68 when the ADP is added to initialize reaction. Curve b: The changes of fluorescence intensity of green QD biosensors without H9 avian influenza virus when the ADP is added to initialize reaction. Curve c:The changes of fluorescence intensity of orange QD biosensors with capturing MHV68 when the ADP is added to initialize reaction. Curve d: The changes of fluorescence intensity of green QD biosensors with capturing H9 avian influenza virus when the ADP is added to initialize reaction [70].

For example, CdTe quantum dots led to an increased effective surface area for immobilization of enzyme and their electrocatalytic activity promoted electron transfer reactions and catalyzed the electro-oxidation of thiocholine, thus amplifying the detection sensitivity [69]. As shown in Figure 7, Deng *et al.* [70] reported that green and orange CdTe QDs can be used as pH-sensitive fluorescent probes, which could monitor the proton (H+) flux driven by ATP synthesis for dual simultaneous and independent detection of viruses on the basis of antibody–antigen reactions.

### 2.5. The Use of Other Nanomaterials in Biosensors

Aside from GNPs, CNTs, magnetic nanoparticles and quantum dots, there are still many other nanomaterials such as metals, metal-oxides [71, 72], polymers and other compounds [73-75], which could be used in biosensors. For example, hollow nanospheres CdS (HS-CdS) [76] were first used to study the direct electrochemical behavior of Hb and the construction of nitrite biosensors. The HS-CdS nanostructure provides a microenvironment around the protein to retain the enzymatic bioactivity.

Metal nanoparticles [77], for example, nano-Cu, with great surface area and high surface energy, are used as electron-conductors and show good catalytic ability to the reduction of H_2_O_2_ [78]. Platinum nanoparticles have also been widely used in biosensors.

**Figure 8.**
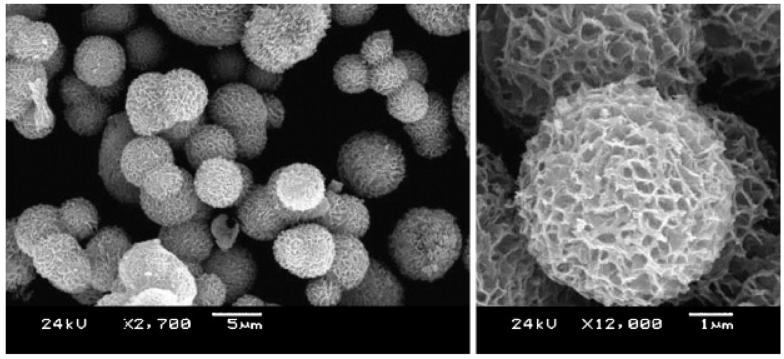
SEM images of as-prepared porous nanosheet-based ZnO microsphere with low (left) and high magnification (right) [83].

Nanoscale metal-oxides have also been widely used in immobilization of proteins and enzymes for bioanalytical applications. For example, metal-oxide-based semiconducting nanowires or nanotubes play an important role on electric, optical, electrochemical and magnetic transducers [79]. Cheng *et al.* [80] reported a nano-TiO_2_ based biosensor for the detection of lactate dehydrogenase (LDH). Waxberry-like nanoscale ZnO balls, as shown in Figure 8, can act as excellent materials for immobilization of enzymes and the rapid electron transfer agent for the fabrication of efficient biosensors due to the wide direct band gap [81, 82]. The porous structure can greatly enhances the active surface area available for protein binding, provide a protective microenvironment for the enzymes to retain their enzymatic stability and activity [83].

Surface functionalized silicon nano-channels with the enzyme urease could detect and quantify urea concentration [84]. The differential conductance of silicon nano-channels can be tuned for optimum performance using the source drain bias voltage, and is sensitive to urea at low concentration. Zhang *et al.* [85] used silicon-on-insulator (SOI) substrate to fabricate the planar type patch clamp ion-channel biosensor, which is suitable for the high throughput screening. The channel current showing the desensitization unique to TRPV1 is measured successfully.

Poly (ethylene-co-glycidyl methacrylate) (PE-co-GMA) nanofibers with abundant active epoxy groups on their surfaces were fabricated through a novel manufacturing process [85,86]. The results demonstrated that the PE-co-GMA nanofibers prepared could be a promising candidate as solid support materials for potential biosensor applications.

## 3. Potential Application of Nanomaterials-Based Biosensors

Although few sensors based on nanomaterials work at all in commercial applications, however, nanomaterial-based biosensors exhibit fascinating prospects. Compared with traditional biosensors, nanomaterial-based biosensors have marked advantages such as enhanced detection sensitivity and specificity, and possess great potential in applications such as the detection of DNA, RNA, proteins, glucose [87], pesticides [88] and other small molecules from clinical samples, food industrial samples, as well as environmental monitoring.

### 3.1. Nanomaterials-Based Biosensors for the Detection of Glucose

The glucose biosensor has been widely used as a clinical indicator of diabetes. Nanoscale materials such as GNPs, CNTs, magnetic nanoparticles [89], Pt nanoparticles [90], Quantum dots, etc. play an important role in glucose sensor performance, fibrous morphology and wrapping of PDDA over MWCNTs result in a high loading of GOx into the electrospun matrix [91]. Pt nanoparticles could be electrodeposited on MWNTs matrix in a simple and robust way. The immobilization of glucose oxidase onto Pt/MWNTs electrode surfaces also could be carried out by chitosan-SiO_2_ gel [92]. The resulting biosensors could be used to determine the glucose levels of serum samples with high sensivity.

### 3.2. Nanomaterials-Based Biosensors for the Detection of DNA and Protein

SsDNA–CNTs probes might be used as optical biosensors to detect specific kinds of DNA oligonucleotides [93]. MWNTs/ZnO/CHIT composite film modified GCE can be used to immobilize ssDNA probes to effectively discriminate different DNA sequences [94, 95]. A biosensor for the detection of deep DNA damage is designed employing the bionanocomposite layer of MWNT in chitosan deposited on a SPCE [96]. The biocomponent represented by double-stranded herring sperm DNA was immobilized on this composite using layer-by-layer coverage to form a robust film. GNPs can also be used to recognize DNA sequences by the interactions of DNA and chemical materials [97]. And for single-stranded DNA, GNPs functionalized with alkanethiol-capped LNA/DNA chimeras in a tail-to-tail hybridization mode could perform excellent [98], and these probes show remarkable discrimination between a complementary target and one containing a single-base mismatch. Nano-SiO_2_/*p*-aminothiophenol (PATP) film was fabricated by self-assembly and electrodeposition methods and was successfully applied to the detection of the PAT gene sequences by a label-free EIS method [99]. Maki *et al.* [100] reported the first nanowire field effect transistor based biosensor which achieves simple and ultra-sensitive electronic DNA methylation detection and avoids complicated bisulfite treatment and PCR amplification. Similarly, using protein–ligand (antigen) interaction properties, protein-nanoparticles based biosensors can realize the ultra-sensitive detection of special protein molecules.

### 3.3. Nanomaterials-Based Biosensors for the Detection of Other Molecules

Liposome-based biosensors have successfully monitored the organophosphorus pesticides such as dichlorvos and paraoxon at very low levels [101]. The nano-sized liposomes provide a suitable environment for the effective stabilization of acethylcholinesterase(AChE) and they can be utilized as fluorescent biosensors. Porins embedded into the lipid membrane allow for the free substrate and pesticide transport into the liposomes. Pesticide concentrations down to 10^−10^ M can be monitored.

By flow injection analysis (FIA), Zhang *et al.* [102] developed a method for the detection of *Escherichia coli* (*E. coli*) using bismuth nanofilm modified GCE. Seo *et al.* [103] constructed a biochip sensor system, consisting of two Ti contact pads and a 150 nm wide Ti nanowell device on LiNbO_3_ substrate. When the bacteria were resistant to the phages (uninfected bacteria), small voltage fluctuations were observed in the nanowell displaying a power spectral density (PSD) of 1/f shape. Medley *et al.* [104] developed a colorimetric assay for the direct detection of diseased cells. This assay uses aptamer-conjugated GNPs to combine the selectivity and affinity of aptamers and the spectroscopic advantages of GNPs. Samples with diseased cells present exhibited a distinct color change while non-target samples did not change the color.

Mitochondrial oxidative stress (MOS) has been hypothesized as one of the earliest insults in diabetes. Some data support the hypothesis that the induction of MOS is more sensitive to hyperglycemia than the induction of the antioxidant response element (ARE). An ARE-GFP vector constructed with nanoparticles was successfully delivered to the eyes by using sub-retinal injection [105]. These data support the use of nanoparticle-delivered biosensors for monitoring the oxidative status of tissues in vivo.

Li *et al.* [106] reported an electrochemical aptamer biosensor for the detection of adenosine based on impedance spectroscopy measurement, which gives not only a label-free but also a reusable platform to make the detection of small molecules simple and convenient. For this method did not rely on the molecule size or the conformational change of the aptamer, it may possess the potential of wider application for different target molecules.

## 4. Challenges and Prospects

In recent years, applications of nanomaterials in biosensors provides novel opportunities for developing a new generation of biosensor technologies. Nanomaterials can improve mechanical, electrochemical, optical and magnetic properties of biosensors, nanomaterial-based biosensors are developing towards single molecule biosensors and high throughput biosensor arrays [107]. However, like any emerging field, they face many challenges. Biological molecules possess special structures and functions, and determining how to fully use the structure and function of nanomaterials and biomolecules to fabricate single molecule multifunctional nanocomposites, nanofilms, and nanoelectrodes, is still a great challenge. The mechanism of interaction between biomolecules and nanomaterials is also not clarified very well yet. How to use these laws and principles of an optimized biosystem for fabricating novel multifunctional or homogenous nanofilms or modifying electrodes is also a great challenge. The processing, characterization, interface problems, availability of high quality nanomaterials, tailoring of nanomateriala, and the mechanisms governing the behavior of these nanoscale composites on the surface of electrodes are also great challenges for the presently existing techniques. For example, how to align nanomaterials such as CNTs in a polymer matrix along identical direction is a great challenge. How to enhance the signal to noise ratio, how to enhance transduction and amplification of the signals, are also great challenges. Future work should concentrate on furtherly clarifying the mechanism of interaction between nanomaterials and biomolecules on the surface of electrodes or nanofilms and using novel properties to fabricate a new generation of biosensors. Nevertheless, nanomaterial-based biosensors show great attractive prospects, which will be broadly applied in clinical diagnosis, food analysis, process control, and environmental monitoring in the near future.
